# Functional variation of *SHP-2* promoter is associated with preterm birth and delayed myelination and motor development in preterm infants

**DOI:** 10.1038/s41598-017-06401-x

**Published:** 2017-07-20

**Authors:** So-Yeon Shim, Hye Jin Jeong, Hyo Jin Park, Eun Young Kwon, Bo Min Kim, Yang Ji Choi, Youn-Hee Choi, Su Jin Cho, Ji Ha Choi, Eun Ae Park

**Affiliations:** 1 Division of Neonatology, Department of Pediatrics, School of Medicine, Ewha Womans University, Seoul, Korea; 2 Neuroscience Research Institute, Gachon University, Incheon, Korea; 3 Department of Pharmacology, Tissue Injury Defense Research Center, School of Medicine, Ewha Womans University, Seoul, Korea; 4 Department of Physiology, Tissue Injury Defense Research Center, School of Medicine, Ewha Womans University, Seoul, Korea

## Abstract

Src homology 2 domain-containing protein tyrosine phosphatase 2 (SHP-2) is a cytoplasmic tyrosine phosphatase that is highly expressed in hematopoietic cells and in the CNS and exerts opposite effects on signal transduction by exerting a neuroprotective or proapoptotic effect. Several mutations of *SHP-2* have been found in children with myeloproliferative disorders or malignant leukemia, and some of these can affect brain development. In the present study, we aimed to identify and functionally characterize genetic variations in *SHP-2* in 72 preterm and 58 full-term infants and to evaluate the effect of the variations on neurodevelopment in preterm infants. Twelve genetic variations were identified. Among them, two variations in the *SHP-2* promoter, g.-317C > T and g.-273G > A, were found to significantly increase promoter activity, and the frequency of g.-273G > A was higher in preterm infants than in full-term infants. Two transcription factors, NF-κB and GABPα, were found to be involved in the transcriptional regulation of *SHP-2* by the two above-mentioned variations. In particular, we found that g.-273G > A was significantly associated with delayed myelination and poor motor development in preterm infants. Our results suggest that a functional promoter variation in *SHP-2* is associated with spontaneous preterm birth itself as well as white matter myelination and neurodevelopment.

## Introduction

Preterm birth is defined as live birth before 37 gestational weeks^1^. Although the mortality rates related to preterm birth have decreased in recent years, neurological impairments in preterm infants remain a problem^2^. Preterm birth is caused due to multiple factors including inflammation, infection, reactive oxygen species (ROS), and genetic factors^3, 4^. These factors also affect immature neuronal cells and result in poor neurological outcome later^5^. To date, several genes such as TNF alpha, IL-1 beta, IL-4, IL-6, and IL-10 have been investigated as candidate genes that modify the risk of preterm labor and perinatal complications^6–8^. Genetic variations in metalloproteinase, endothelial nitric oxide synthetase, superoxide dismutase, and catalase also have been suggested as risk factors for preterm birth^9–12^. However, the precise mechanism underlying the regulation of these genes remains obscure.

Src homology 2 domain-containing protein tyrosine phosphatase 2 (SHP-2) (also known as PTPN11) is a cytoplasmic tyrosine phosphatase that is widely expressed at high levels in hematopoietic cells and in the central nervous system (CNS)^13, 14^. SHP-2 is involved in neuroprotection in response to ischemic brain injury, and SHP-2 inhibition leads to reduced survival and increased programmed cell death of primary cultured neurons during nitric oxide exposure^15–17^. SHP-2 also functions as an important protein component of the raft-mediated signaling pathway and as a key regulator of a signaling cascade upon ROS-induced oxidative stress^18, 19^. Recently, several mutations in *SHP-2* were found in children with diverse myeloproliferative disorders or malignant leukemia such as juvenile myelomonocytic leukemia (JMML), myelodysplastic syndrome, B cell acute lymphocytic leukemia, and acute myeloid leukemia^20–23^. In particular, the two most common mutations associated with JMML are known to cause a gain of function (GOF)^23^. Noonan syndrome, which is a frequent genetic disease with an estimated prevalence of approximately 1/2,000 living births, is caused by genetic variations in genes involved in Ras-Erk signaling, including *SHP-2* ^24^. Interestingly, a nonsynonymous GOF variation in *SHP-2*, D61G, is associated with aspects of neurodevelopment such as spatial learning and memory deficits in Noonan syndrome^25^. These results support a disease-associated function for SHP-2 against leukemia and brain development.

Therefore, we hypothesized that genetic variations in *SHP-2* affect spontaneous preterm birth by regulating cytokines and a signaling pathway under ROS-induced oxidative stress. Furthermore, we reckoned that *SHP-2* variations might also be associated with neurodevelopment in preterm infants, because SHP-2 is highly expressed in the CNS and works as a regulator of Ras-Erk signaling involved in neurodevelopment. To test our hypothesis, we compared genetic variations in *SHP-2* between preterm and full-term infants and functionally characterized each variation using various *in vitro* assays. To evaluate the effect of the genetic variations in *SHP-2* on neurodevelopment in preterm infants, we further analyzed the degree of myelination according to the functional genetic variation in *SHP-2* using tract-based spatial statistics (TBSS) and assessed neurodevelopment using the Bayley Scale of Infant and Toddler Development, third edition (Bayley-III), in preterm infants at 18–22 months of corrected age.

## Results

### Study population

The characteristics of the study subjects are shown in Supplementary Fig. 1. We enrolled 72 preterm infants and 58 full-term infants for the genetic analysis of *SHP-2*. Blood samples from all the infants were obtained in the neonatal period during hospitalization or at infancy when they visited the outpatient clinic. Table 1 shows the demographic data of the study subjects. Among the mothers of the 72 preterm infants, 52 mothers were pregnant for the first time. Among the remaining 20 mothers, only one mother had a medical history of previous preterm birth. Among the 72 preterm infants, nine died during hospitalization, and 17 were lost during follow-up. In the nine mortality cases, there were no congenital malformations such as congenital heart disease or brain or other organ malformation, and none of the cases showed abnormal appearance. The remaining 46 preterm infants were re-visited at 18–22 months of corrected age for assessment of neurological development.

**Table 1:** Demographic data of study population.

Variable	Preterm infant, n (%)	Full-term infant, n (%)	Preterm infant, mean ± SD	Full-term infant, mean ± SD	*P*-value
Total number	72	58			
Gestational age (weeks)			28^+5^ ± 3^+2^	38^+5^ ± 1^+1^	<0.001
Birth weight (g)			1174 ± 492	3067 ± 610	<0.001
Male	36 (50.0)	30 (51.7)			0.845
Cesarean section	44 (61.1)	22 (37.9)			0.009
BPD	31 (43.1)	0			<0.001
IVH	46 (63.9)	3 (5.2)			<0.001
PVL	9 (12.5)	0			0.005
Sepsis	6 (8.3)	0			0.024
Mortality	9 (12.5)	0			0.005

BPD, bronchopulmonary dysplasia; IVH, intraventricular hemorrhage; PVL, periventricular leukomalacia. n, number.

### Genetic variations in *SHP-2* in the preterm and full-term infants

To investigate the effect of *SHP-2* variations on susceptibility to preterm birth, we identified genetic variations in *SHP-2* in 130 preterm or full-term infants through direct sequencing or genotyping and compared the frequency of each variation between the preterm and full-term infants. Table 2 shows the frequencies of *SHP-2* variations in our study subjects. Twelve genetic variations including two variations in the promoter region and one nonsynonymous variation were identified. Among them, four intron variations and one nonsynonymous variation were first identified in this study. Table 3 lists the comparison of the frequency of each variation between the two groups. The frequency of one of the promoter variations, g.-273G > A, was higher in the preterm infants than in the full-term infants (*P* = 0.025). The frequency of the other variations were similar between the two groups.

**Table 2:** Frequency of *SHP-2* genetic variations in preterm or full-term infants.

Variation (rs number)	Minor allele	Minor allele frequency		Variation (rs number)	Minor allele	Minor allele frequency	
		Preterm	Full-term			Preterm	Full-term
g.-317C > T (rs373537430)	T	0.007	0.000	IVS11 + 20 C > T (rs184743462)	T	0.007	0.009
g.-273G > A (rs58805176)	A	0.292	0.198	IVS11 − 75T > C	C	0.007	0.000
IVS1 + 21 C > G	G	0.028	0.009	IVS13 − 95C > T (rs3741983)	C	0.146	0.207
IVS2 + 143 G > A	A	0.000	0.009	P559S	T	0.007	0.000
IVS4 − 19C > T	T	0.007	0.000	IVS14 − 146G > A (rs4767860)	G	0.430	0.397
IVS10 − 63G > A (rs141247150)	A	0.007	0.000	1960T > C (rs17849094)	C	0.021	0.009

Data was obtained from DNA samples from 72 preterm infants and 58 full-term infants. Nucleotide location numbers were assigned from the translational start site based on the *SHP-2* mRNA sequence (GenBank accession number; NM_002834.3).

**Table 3:** Comparison of *SHP-2* genetic variations between preterm and full-term infants.

Variation	Zygosity	Preterm infant, n (%)	Full-term infant, n (%)	*P*-value^a^
g.-317C > T	C/C	71 (98.6)	58 (100.0)	1.000
	C/T	1 (1.4)	0	
	T/T	0	0	
g.-273G > A	G/G	33 (45.8)	38 (65.5)	0.025
	G/A	36 (50.0)	17 (29.3)	
	A/A	3 (4.2)	3 (5.2)	
IVS1 + 21 C > G	C/C	68 (94.4)	57 (98.3)	0.380
	C/G	4 (5.6)	1 (1.7)	
	G/G	0	0	
IVS2 + 143 G > A	G/G	72 (100.0)	57 (98.3)	0.446
	G/A	0	1 (1.7)	
	A/A	0	0	
IVS4 − 19C > T	C/C	71 (98.6)	58 (100.0)	1.000
	C/T	1 (1.4)	0	
	T/T	0	0	
IVS10 − 63G > A	G/G	71 (98.6)	58 (100.0)	1.000
	G/A	1 (1.4)	0	
	A/A	0	0	
IVS11 + 20 C > T	C/C	71 (98.6)	57 (98.3)	1.000
	C/T	1 (1.4)	1 (1.7)	
	T/T	0	0	
IVS11 − 75T > C	T/T	71 (98.6)	58 (100.0)	1.000
	T/C	1 (1.4)	0	
	C/C	0	0	
IVS13 − 95C > T	T/T	52 (72.2)	37 (63.8)	0.304
	T/C	19 (26.4)	18 (31.0)	
	C/C	1 (1.4)	3 (5.2)	
P559S (c.1675C > T)	C/C	71 (98.6)	58 (100.0)	1.000
	C/T	1 (1.4)	0	
	T/T	0	0	
IVS14 − 146G > A^b^	A/A	20 (28.2)	23 (39.6)	0.169
	A/G	41 (57.7)	24 (41.4)	
	G/G	10 (14.1)	11 (19.0)	
1960T > C^b^	T/T	68 (95.8)	57 (98.3)	0.627
	T/C	3 (4.2)	1 (1.7)	
	C/C	0	0	

^a^The *P-*values were obtained using dominant model. ^b^The genotype data could not be obtained in a preterm infant because of a sequencing failure.

### Effect of *SHP-2* promoter variations on the promoter activity of the gene

**Figure 1:**
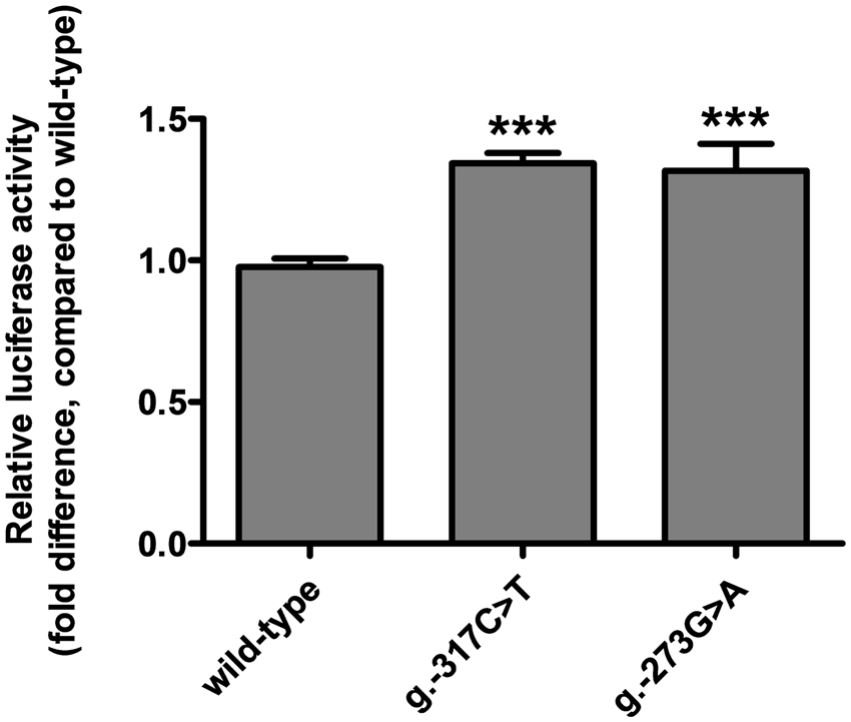
Luciferase activities of the vectors containing *SHP-2* promoter variations. The luciferase activities were measured 48 h after transfection of the reporter vectors containing the wild-type *SHP-2* promoter sequence or its genetic variations into HCT-116 cells. Then, the luciferase activity of each vector was compared with that of the wild-type. The data (mean ± SD) were obtained from triplicate wells. ***P < 0.001 vs. wild-type.

**Figure 2:**
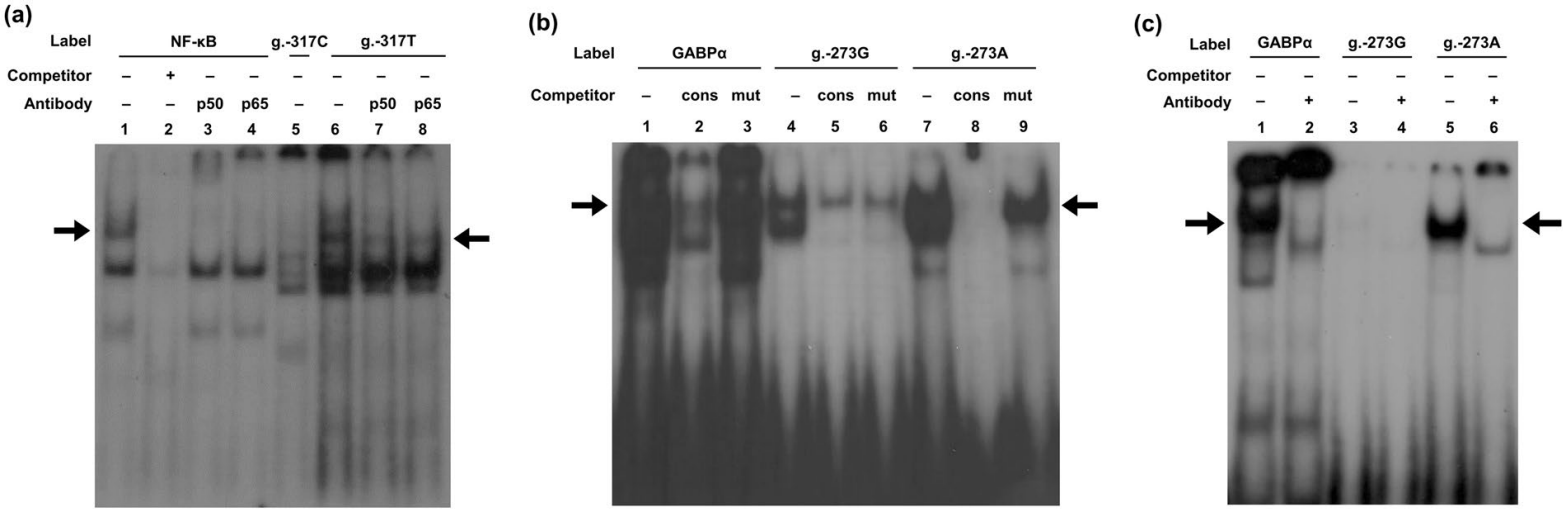
EMSAs for identification of the interaction between *SHP-2* promoter variations and transcription factors. (**a**) ^32^P-labeled oligonucleotides (lanes 1–3, NF-κB consensus; lanes 4–6, g.-317C wild-type; lanes 7–9, g.-317T variant) were incubated with nuclear protein extracts from HCT-116 cells. A competition assay and a supershift assay were conducted using 100-fold molar excess of unlabeled NF-κB consensus oligonucleotides (lane 2) and antibodies against NF-κB (lanes 3, 4, 7, and 8), respectively. (**b**) ^32^P-labeled oligonucleotides (lanes 1–3, GABPα consensus; lanes 4-6, g.-273G wild-type; lanes 7–9, g.-273A variant) were incubated with nuclear protein extracts. A competition assay was performed using 100-fold molar excess of unlabeled GABPα consensus (cons, lanes 2, 5, and 8) or mutant (mut, lanes 3, 6, and 9) oligonucleotides. (**c**) A supershift assay was performed with the antibody against GABPα (lanes 2, 4, and 6). The arrows indicate DNA-protein complexes.

To the best of our knowledge, there has been no report on the functional characterization of genetic variations in the *SHP-2* promoter. Therefore, we investigated the effect of *SHP-2* promoter variations on the promoter activity of the gene by measuring the luciferase activity of the reporter vectors containing wild-type or mutant *SHP-2* promoter. We observed that two variations, g.-317C > T and g.-273G > A, significantly increased the promoter activity of *SHP-2* by 36.7% and 34.0%, respectively, compared to that of the wild-type (Fig. 1). We then predicted the potential transcription factors that could bind to the *SHP-2* promoter near the two above-mentioned variations using MatInspector (Genomatrix Software GmbH, Munich, Germany) in order to investigate the mechanism underlying the transcriptional regulation of the *SHP-2* promoter. Two transcription factors, nuclear factor kappa-light-chain-enhancer of activated B cells (NF-κB) and GA-binding protein alpha (GABPα), were predicted to bind to the two variations, g.-317C > T and g.-273G > A, respectively, and there was a large difference in the binding affinity between the wild-type and variant sequences. To validate our prediction, we conducted electrophoretic mobility shift assays (EMSAs). First, we confirmed the position of DNA-protein complexes consisting of NF-κB consensus oligonucleotides and nuclear extracts through a competition assay and a supershift assay (lanes 1–4, Fig. 2a). Through binding reaction of wild-type (g.-317C) or variant (g.-317T) oligonucleotides with the nuclear protein extracts, we observed that NF-κB could bind to the *SHP-2* promoter near g.-317C > T and that this transcription factor bound to the variant more strongly than to the wild-type (lanes 5–8, Fig. 2a). Next, we confirmed the position of DNA-protein complexes consisting of GABPα consensus oligonucleotides and nuclear protein extracts through a competition assay (lanes 1–3, Fig. 2b) and found that GABPα could bind to the *SHP-2* promoter near g.-273G > A and that the binding affinity for the *SHP-2* promoter was much stronger in the presence of the variant, g.-273A (lanes 4 and 7, Fig. 2b). Figure 2c shows the result of a supershift assay conducted using a GABPα antibody. A supershift in the presence of an antibody confirmed that GABPα was present in the DNA-protein complexes (lanes 2, 4, and 6, Fig. 2c).

### Effect of a *SHP-2* nonsynonymous variation on SHP-2 expression

**Figure 3:**
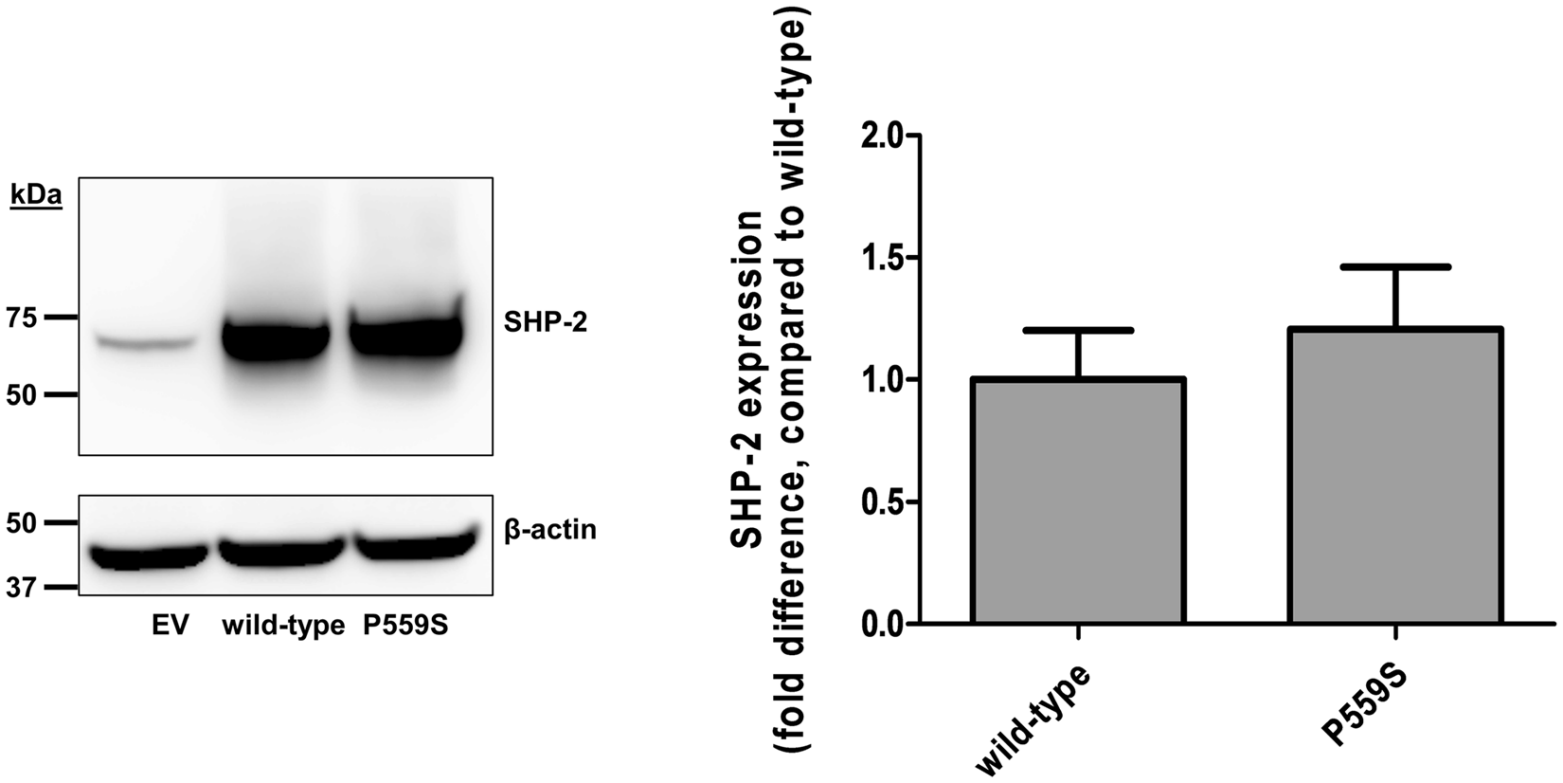
Effect of a variation, P566S, on SHP-2 expression. SHP-2 expression was investigated using immunoblotting after transfection of wild-type *SHP-2* or P566S variation vectors into HCT-116 cells. Data shown represent mean ± SD obtained from three independent experiments and analyzed by Student’s two-tailed *t*-test. EV; empty vector.

In general, a nonsynonymous variation, which causes a change in amino acids, could affect the expression or function of a protein. In the current study, one nonsynonymous variation, P559S, was found in a healthy preterm infant. To investigate the effect of this variation on SHP-2 expression, we performed immunoblotting assays and found that SHP-2 expression was increased by 20.7% in the presence of P559S, although the difference was not statistically significant (*P* = 0.765, Fig. 3).

### Effect of the functional promoter variation, g.-273G > A, on myelination in preterm infants

**Figure 4:**
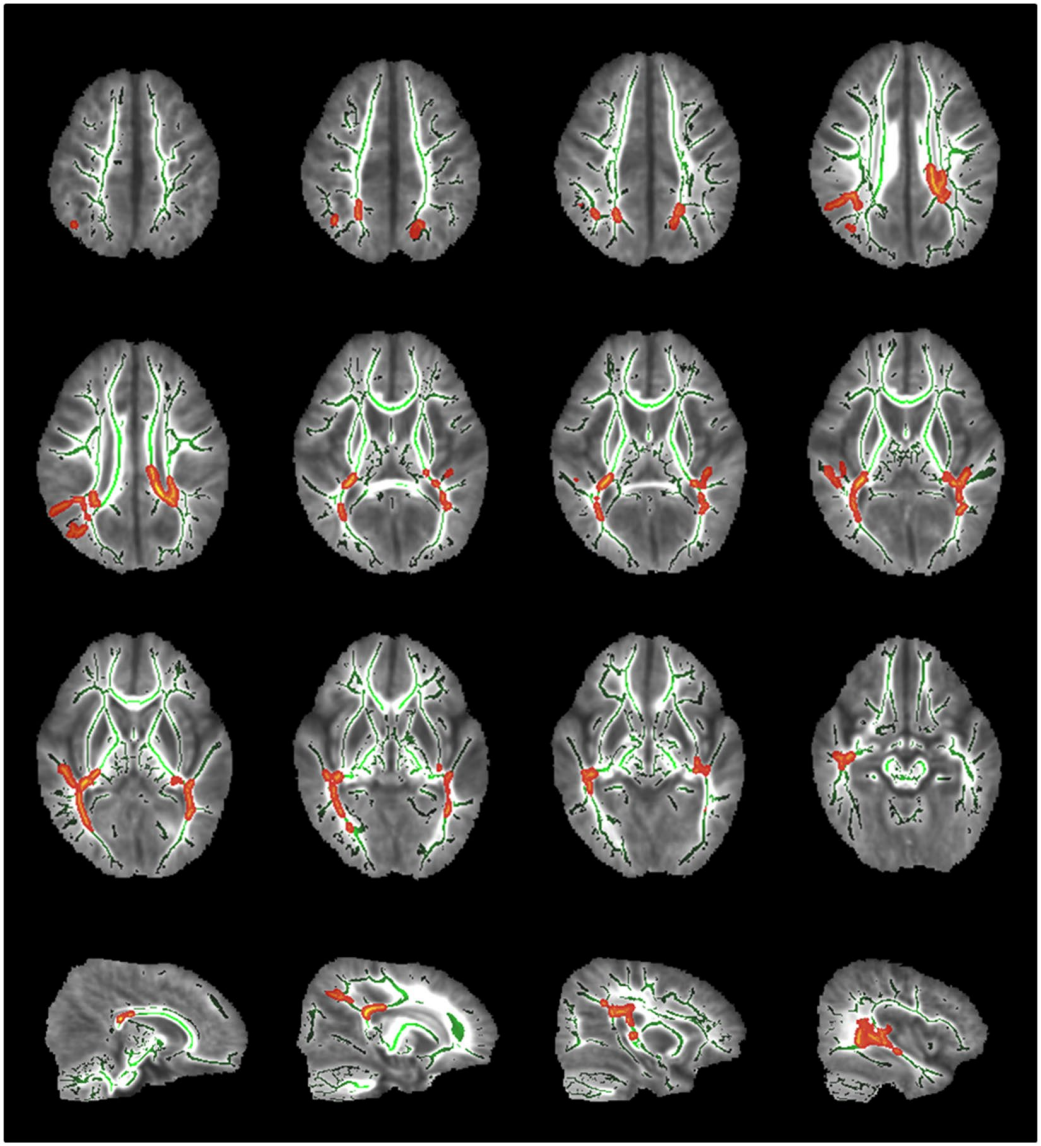
TBSS results. Comparisons of the mean FA maps of preterm infants at 18–22 months of corrected age between the *SHP-2* variant and control groups. Mean FA skeleton is shown in green, and areas with significant differences between the two groups are shown in red-yellow (*P* < 0.05). The FA value in the corpus callosum, posterior limb of internal capsule, and optic radiation was lower in the variant group.

To examine the effect of the functional variation in the *SHP-2* promoter on myelination in preterm infants, we divided the 46 preterm infants into two groups: a variant group (n = 23) and a control group (n = 23). The variant group consisted of subjects heterozygous or homozygous for g.-273G > A, and the control group consisted of the remaining infants. Because the infant with the other functional *SHP-2* promoter variation, g.-317C > T, had passed away during hospitalization, this variation was not considered in our analysis. Tract-based spatial statistics (TBSS) revealed that the fractional anisotropy (FA) value in the corpus callosum, posterior limb of internal capsule (PLIC), and optic radiation was significantly lower in the variant (g.-273A) group than in the control (g.-273G) group (*P* < 0.05) (Fig. 4). These immature myelinations in the variant group spread around the parietal, frontal, and temporal regions (Supplementary Table S1).

### Effect of the functional promoter variation, g.-273G > A, on neurodevelopment in preterm infants

We then examined the effect of the functional variation in the *SHP-2* promoter on neurodevelopment in preterm infants. Motor, cognitive, language, and social development were assessed at 18–22 months of corrected age in the 46 preterm infants, and data were analyzed according to the g.-273G > A variation. Grossly, the preterm infants in the variant group showed lower scores than those in the control group (Table 4). In particular, the motor composite score was significantly lower in the preterm infants with the *SHP-2* promoter variation (*P* = 0.027). Table 5 shows the proportion of delayed development in each developmental subscore; in all the subscores, the proportion of delayed development was higher in the variant group. Delayed motor development accounted for approximately 40% in the variant group, which was significantly different compared to that in the control group (*P* = 0.004).

**Table 4:** Neurodevelopmental scores of Bayle III in preterm infants according to the g.-273G > A variation at 18–22 months of corrected age.

Neurodevelopmental score	Variant (n = 23), mean ± SD	Control (n = 23), mean ± SD	*P*-value
Corrected age at assessment (months)	19.1 ± 3.3	18.7 ± 2.3	0.653
Motor composite score	89.1 ± 16.1	100.4 ± 17.2	0.027
Cognitive composite score	92.4 ± 16.9	93.7 ± 13.6	0.767
Language composite score	84.4 ± 14.5	90.8 ± 11.9	0.112
Social composite score	94.6 ± 16.4	96.5 ± 19.4	0.733

**Table 5:** The proportion of developmental delay in preterm infants according to the g.-273G > A variation at 18–22 months of corrected age.

Developmental delay	Variant (n = 23), n (%)	Control (n = 23), n (%)	*P*-value
Motor composite	9 (39.1)	1 (4.3)	0.004
Cognitive composite	6 (26.1)	2 (8.7)	0.120
Language composite	12 (52.2)	6 (26.1)	0.070
Social composite	4 (17.4)	4 (17.4)	1.000

## Discussion

SHP-2 is ubiquitously expressed in mammalian tissues with high levels of expression in hematopoietic cells and in the CNS and has been shown to be essential for organ development and hematopoiesis^26^. Dysregulation of SHP-2 function or expression has been implicated in the pathogenesis of various human diseases^26^. In spite of the proven clinical importance of SHP-2, there has been no study examining the association between genetic variations in *SHP-2* and preterm births. In the present study, we observed that two genetic variations in the *SHP-2* promoter, g.-317C > T and g.-273G > A, resulted in significantly increased promoter activity and that the frequency of g.-273G > A was higher in preterm infants than in full-term infants. In the 1,000 genome project (https://www.ncbi.nlm.nih.gov/variation/tools/1000genomes/), the mean minor allele frequency of g.-273G > A was found to be 0.143. However, the minor allele frequency of this variation was different in different ethnicities; in general, the minor allele frequency was higher in Asians than in other populations such as Africans or Europeans (Supplementary Table S2). In our study population, the minor allele frequency of g.-273G > A was high; in particular, it was higher in the preterm infants (0.292) than in the full-term infants (0.198). In addition, we found that two transcription factors, NF-κB and GABPα, are involved in the transcriptional regulation of *SHP-2* by the two above-mentioned variations.

Preterm birth and its associated complications such as brain injury have been known to be caused by multiple pathogenic factors. Maternal or fetal infection and inflammation can induce the release of cytokines such as IL-1, IL-8, IL-10, and TNF, chemokines, and platelet-activating factors^27^. These responses can induce preterm labor^3, 4^ and increase ROS production, resulting in the injury of immature oligodendrocytes^5^. The deficient antioxidant capacity of preterm infants further aggravates the insult caused by ROS. Apoptosis of immature oligodendrocytes by ROS is the main pathology of brain injury in preterm infants^5^.

While other protein tyrosine phosphatases are widely accepted as negative regulators of signaling events, SHP -2 can act as either a positive or a negative regulator in signaling cascades in a cell type-specific and stimulant-specific manner^28^. SHP-2 is one of the rare protein tyrosine phosphatases that display dual function in signal transduction, showing both a neuroprotective effect and a proapoptotic effect by promoting caspase activation^15, 16, 19^. Since previous studies have reported that SHP-2 plays key roles in various cellular processes including proliferation, survival, differentiation, and metabolism^26^ and because SHP-2 is known to be highly expressed in specific brain regions including the cortex, cerebellum, and hippocampus^13^, we compared brain diffusion tensor image (DTI) data in the preterm infants according to the *SHP-2* promoter variation, g.-273G > A, and found that myelination of the corpus callosum, PLIC, and optic radiation was significantly delayed in the variant group than in the control group. TBSS is an observer-independent tool used for analyzing DTI data^29^. Using TBSS, brain maturation and tract organization can be detected across all white matter areas in the absence of apparent brain abnormalities in preterm infants^30, 31^. In general, FA increases with age, which is believed to reflect white matter maturations such as fiber coherence, axonal density, and myelination^32^. The corpus callosum, PLIC, and optic radiation have been known to be involved in visual motor function^33–35^. Our finding of delayed myelination in these areas and poorer motor function in the same subjects is consistent with the results of the above-mentioned previous studies. Another previous study reported that gain of function (GOF) genetic variations in *SHP-2* resulting in increased numbers of oligodendrocyte progenitor cells and MAPK activity negatively influence myelination by inducing abnormal myelination or resulting in fewer myelinated axons in white matter^36^. This study also supports our results that indicate that GOF variations in *SHP-2* can negatively affect myelination.

As mentioned above, SHP-2 is highly expressed in hematopoietic cells and is essential for hematopoiesis^26^. Therefore, we also investigated whether g.-273G > A can affect hematologic features in the preterm infants. Among the 72 preterm infants, seven had disseminated intravascular coagulation (DIC), which is characterized by systemic activation of pathways leading to and regulating coagulation. DIC may cause organ failure with concomitant consumption of platelets and coagulation factors, which may result in clinical bleeding^37^. However, we found no statistically significant difference in the occurrence of DIC between the g.-273G > A variant and wild-type groups [5 infants (12.8%) vs. 2 infants (6.1%), *P* = 0.442]. We also did not find any other abnormal hematological features in our study population. Therefore, we concluded that there was no significant difference in the hematologic features between the preterm infants with g.-273G > A and those without this variation in our study.

Our study has a few limitations. First, the sample size was not large enough to reach sufficient statistical power. However, the strength of our study lies in the fact that we identified genetic variations in our study population and then investigated the effect of each variation on gene function at the molecular level. Moreover, we identified that the functional *SHP-2* promoter variation is associated with myelination and neurodevelopment by using advanced MRI and neurodevelopmental assessment tools. The second limitation of our study is that we could not measure SHP-2 expression levels in the infants with functional variations in the *SHP-2* promoter. Finally, neurodevelopmental assessment was performed at 18–22 months of corrected age. The evaluation during this period may not be enough to assess cognitive or social development^38^. Follow-up assessment might provide more information regarding cognitive and social development associated with *SHP-2* variations in our population.

In summary, in the present study, we identified functional variations in the promoter region of *SHP-2* and found that one GOF variation in the *SHP-2* promoter is associated with spontaneous preterm birth itself as well as delayed myelination and poor motor development in preterm infants. To the bets of our knowledge, this is the first study examining the relationship between genetic variations in the *SHP-2* promoter and spontaneous preterm birth or brain development. Because SHP-2 is linked with cytokines, ROS production, and apoptosis, which have been known to be the main pathologies of preterm birth and its associated complications such as brain injury, SHP-2 might be an important molecule with regard to preterm births. Further studies with larger sample sizes are necessary to confirm our results.

## Methods

### Genetic analysis of *SHP-2*

This study was reviewed and approved by the Institutional Review Board of the Ewha Medical Center, Seoul, Korea. All the experiments were performed in accordance with relevant guidelines and regulations of Institutional Review Board of the Ewha Medical Center. Seventy-two preterm infants (≤35 gestational weeks at birth, preterm group) and 58 healthy full-term infants (≥37 gestational weeks at birth, full-term group) were included in this study. Written informed consent was obtained from the legal representatives of all participants prior to enrollment. To identify the genetic variations in *SHP-2*, the promoter (up to −2,168 bp from the translation start site) or coding (all exons and exon-intron boundaries) regions of *SHP-2* were amplified by PCR using DNA samples obtained from the preterm and full-term infants. The PCR conditions were as follows: initial denaturation at 94 °C for 5 min, followed by 35 cycles of denaturation at 94 °C for 30 s, annealing at 55**–**65 °C for 30 s, initial extension at 72 °C for 30–60 s, and final extension at 72 °C for 10 min. Then, the PCR products were purified using a MultiScreen384-PCR Filter Plate (Milipore, Billerica, MA, USA) and sequenced using a BigDye Terminator Cycle Sequencing Kit and an ABI 3730xl automated sequencer (Applied Biosystems, Foster City, CA, USA). Mutation analyses were performed using Phred, Phrap, Consed, Polyphred 5.04 software (http://droog.mbt.washington.edu/PolyPhred.html).

### Construction of vectors containing the *SHP-2* promoter or coding regions

To construct a luciferase reporter vector containing the promoter region of the *SHP-2* wild-type sequence, a 448-bp region of the *SHP-2* promoter was amplified using PCR with the primers listed in Supplementary Table S3 and a genomic DNA sample obtained from an individual with a wild-type sequence. This PCR product was then inserted into the pGL4.11 [luc2P] vector (Promega Corporation, Madison, WI, USA). For a vector containing the wild-type *SHP-2* coding region, the pCMV-SHP-2 wild-type vector (Addgene plasmid #8381), a kind gift from Ben Neel, was obtained from Addgene (Addgene, Cambridge, MA, USA). The mutant vectors containing genetic variations in the promoter or coding regions were generated using QuikChange® II Site-Directed Mutagenesis Kit (Agilent Technologies, Santa Clara, CA, USA) with primers shown in Supplementary Table S3. The sequences of all the constructs were confirmed by direct sequencing.

### Measurement of the promoter activity of wild-type *SHP-2* or its variations

Forty-eight hours after the transfection of the reporter vectors into HCT-116 cells using Lipofectamine LTX and Plus reagents (Life Technologies, Carlsbad, CA, USA), the promoter activity of each vector was measured using a Dual-luciferase® reporter assay system and a Glomax 96-well plate luminometer (Promega, Fitchburg, WI, USA) by following the manufacturer’s instructions.

### EMSA

EMSAs were conducted as described previously^39^. First, the binding reaction was performed by incubating ^32^P-labeled oligonucleotides (1 × 10^5^ counts/min) with 10–20 µg of nuclear protein extracts obtained from HCT-116 cells for 30 min at room temperature. For the competition assay, the unlabeled NF-κB consensus or GABPα consensus or mutant oligonucleotides were added in 100-fold molar excess prior to the binding reaction. The supershift assay was performed using three kinds of antibodies, NF-κB p50 (sc-7178X, Santa Cruz Biotechnology, Santa Cruz, CA, USA), NF-κB p65 (sc-372X, Santa Cruz Biotechnology), and GABPα (sc-22810X, Santa Cruz Biotechnology). Then, each sample was electrophoresed for 90 min at 80 V, and the dried gel was exposed to a CP-BU film (Agfa, Mortsel, Belgium) for 16 h at −80 °C to detect the signal. The intensity of each band was measured using ImageJ software (National Institutes of Health, Bethesda, MD, USA). Supplementary Table S3 lists the oligonucleotides used in the EMSAs.

### Immunoblotting

Forty-eight hours after the transfection of the wild-type *SHP-2* or *SHP-2* mutant-containing vectors into HCT-116 cells using Lipofectamine LTX and Plus reagents, immunoblotting was performed using a mouse anti-SHP-2 antibody (BD Biosciences, San Jose, CA, USA) or a goat anti-β-actin antibody (Santa Cruz Biotechnology). The intensity of each band was measured using ImageJ software.

### Imaging data acquisition and analysis

DTIs were obtained using 3-tesla MRI (Phillips, Foster City, CA, USA) at 18–22 months of corrected age as described previously^31, 40^. Before obtaining the DTIs, 3D MPRAGE images and high-resolution T1- and T2-weighted images were obtained. DTI sequence parameters were substituted as follows: b = 0 and 800 s/mm^2^ and TR/TE = 10,100/76 ms. The scanning times for the DTI sequences were 7 min and 36 s at TEA and 10 min and 18 s. TBSS was performed as described in our previous study^31^ to compare the degree of myelination between the *SHP-2* promoter variant (g.-273A) and control (g.-273G) groups. Briefly, all the FA images were aligned to a target in a common space using an optimized TBSS protocol for neonates^41^. Voxels with *P* < 0.05 (corrected for multiple comparisons) were considered significantly different.

### Neurodevelopmental assessment

Neurodevelopment was assessed at 18–22 months of corrected age by trained psychologists. Development was assessed using Bayley-III, which provided a motor composite score, a cognitive composite score, a language composite score, and a social composite score. For all the subscores, cut-off points of <85 (1 standard deviation (SD) below normative mean) and <70 (2 SD below normative mean) were used to identify mild to moderate and severe delay, respectively. In the present study, delayed development included mild to moderate and severe delay.

### Statistical analysis

Statistical analyses were performed using the SPSS v.23.0 software package (IBM Corporation, Armonk, NY, USA). The data shown in the luciferase assay and immunoblotting represent mean ± SD from more than three separate experiments. *P* values for comparison of the frequencies of genetic variations between the preterm and full-term infants were calculated using the χ^2^-test. *P* values for the luciferase assay and immunoblotting were calculated using one-way analysis of variance, followed by Dunnett’s two-tailed test and Student’s two-tailed t-test, respectively. The comparisons of neurodevelopmental assessment results between the variant and control groups were performed using the χ^2^-test for categorical variables and Student’s two-tailed t-test for continuous variables. *P* values < 0.05 were considered to indicate statistical significance.

## Electronic supplementary material

Supplementary Information

## References

[1] Monangi NK, Brockway HM, House M, Zhang G, Muglia LJ (2015). The genetics of preterm birth: Progress and promise. Semin Perinatol.

[2] Woodward LJ (2009). Very preterm children show impairments across multiple neurodevelopmental domains by age 4 years. Arch Dis Child Fetal Neonatal Ed.

[3] de Andrade Ramos BR, Witkin SS (2016). The influence of oxidative stress and autophagy cross regulation on pregnancy outcome. Cell Stress Chaperones.

[4] Varner MW, Esplin MS (2005). Current understanding of genetic factors in preterm birth. BJOG.

[5] Volpe JJ (2001). Neurobiology of periventricular leukomalacia in the premature infant. Pediatr Res.

[6] Langmia IM, Apalasamy YD, Omar SZ, Mohamed Z (2016). Impact of IL1B gene polymorphisms and interleukin 1B levels on susceptibility to spontaneous preterm birth. Pharmacogenet Genomics.

[7] Huusko JM (2014). A study of genes encoding cytokines (IL6, IL10, TNF), cytokine receptors (IL6R, IL6ST), and glucocorticoid receptor (NR3C1) and susceptibility to bronchopulmonary dysplasia. BMC Med Genet.

[8] Baier RJ (2006). Genetics of perinatal brain injury in the preterm infant. Front Biosci.

[9] Frey HA (2016). Genetic variation associated with preterm birth in African-American women. Am J Obstet Gynecol.

[10] Vannemreddy P, Notarianni C, Yanamandra K, Napper D, Bocchini J (2010). Is an endothelial nitric oxide synthase gene mutation a risk factor in the origin of intraventricular hemorrhage?. Neurosurg Focus.

[11] Giusti B (2012). Genetic polymorphisms of antioxidant enzymes as risk factors for oxidative stress-associated complications in preterm infants. Free Radic Res.

[12] Poggi C (2015). Genetic Contributions to the Development of Complications in Preterm Newborns. PLoS One.

[13] Suzuki T, Matozaki T, Mizoguchi A, Kasuga M (1995). Localization and subcellular distribution of SH-PTP2, a protein-tyrosine phosphatase with Src homology-2 domains, in rat brain. Biochem Biophys Res Commun.

[14] Servidei T (1998). The protein tyrosine phosphatase SHP-2 is expressed in glial and neuronal progenitor cells, postmitotic neurons and reactive astrocytes. Neuroscience.

[15] Park H, Ahn KJ, Lee Kang J, Choi YH (2015). Protein-protein interaction between caveolin-1 and SHP-2 is dependent on the N-SH2 domain of SHP-2. BMB Rep.

[16] Morales LD (2014). Protein tyrosine phosphatases PTP-1B, SHP-2, and PTEN facilitate Rb/E2F-associated apoptotic signaling. PLoS One.

[17] Zhang X (2012). Loss of Shp2 in alveoli epithelia induces deregulated surfactant homeostasis, resulting in spontaneous pulmonary fibrosis. FASEB J.

[18] Yun JH (2011). Caveolin-1 is involved in reactive oxygen species-induced SHP-2 activation in astrocytes. Exp Mol Med.

[19] Jo A (2014). SHP-2 binds to caveolin-1 and regulates Src activity via competitive inhibition of CSK in response to H2O2 in astrocytes. PLoS One.

[20] Loh ML (2004). PTPN11 mutations in pediatric patients with acute myeloid leukemia: results from the Children’s Cancer Group. Leukemia.

[21] Loh ML (2004). Mutations in PTPN11 implicate the SHP-2 phosphatase in leukemogenesis. Blood.

[22] Tartaglia M (2004). Genetic evidence for lineage-related and differentiation stage-related contribution of somatic PTPN11 mutations to leukemogenesis in childhood acute leukemia. Blood.

[23] Tartaglia M (2003). Somatic mutations in PTPN11 in juvenile myelomonocytic leukemia, myelodysplastic syndromes and acute myeloid leukemia. Nat Genet.

[24] Tartaglia M (2001). Mutations in PTPN11, encoding the protein tyrosine phosphatase SHP-2, cause Noonan syndrome. Nat Genet.

[25] Lee YS (2014). Mechanism and treatment for learning and memory deficits in mouse models of Noonan syndrome. Nat Neurosci.

[26] Tajan M, de R Serra A, Valet P, Edouard T, Yart A (2015). SHP2 sails from physiology to pathology. Eur J Med Genet.

[27] Manuck TA (2016). The genomics of prematurity in an era of more precise clinical phenotyping: A review. Semin Fetal Neonatal Med.

[28] Dance M, Montagner A, Salles JP, Yart A, Raynal P (2008). The molecular functions of Shp2 in the Ras/Mitogen-activated protein kinase (ERK1/2) pathway. Cell Signal.

[29] Smith SM (2006). Tract-based spatial statistics: voxelwise analysis of multi-subject diffusion data. Neuroimage.

[30] Shim SY (2012). Altered microstructure of white matter except the corpus callosum is independent of prematurity. Neonatology.

[31] Shim SY (2014). Serial diffusion tensor images during infancy and their relationship to neuromotor outcomes in preterm infants. Neonatology.

[32] Dubois J, Hertz-Pannier L, Dehaene-Lambertz G, Cointepas Y, Le Bihan D (2006). Assessment of the early organization and maturation of infants’ cerebral white matter fiber bundles: a feasibility study using quantitative diffusion tensor imaging and tractography. Neuroimage.

[33] Thompson DK (2012). Corpus callosum alterations in very preterm infants: perinatal correlates and 2 year neurodevelopmental outcomes. Neuroimage.

[34] Bassi L (2008). Probabilistic diffusion tractography of the optic radiations and visual function in preterm infants at term equivalent age. Brain.

[35] Rose J (2015). Neonatal brain microstructure correlates of neurodevelopment and gait in preterm children 18-22 mo of age: an MRI and DTI study. Pediatr Res.

[36] Ehrman LA (2014). The protein tyrosine phosphatase Shp2 is required for the generation of oligodendrocyte progenitor cells and myelination in the mouse telencephalon. J Neurosci.

[37] Levi M, Toh CH, Thachil J, Watson HG (2009). Guidelines for the diagnosis and management of disseminated intravascular coagulation. British Committee for Standards in Haematology. Br J Haematol.

[38] Latal B (2009). Prediction of neurodevelopmental outcome after preterm birth. Pediatr Neurol.

[39] Jang GH, Kim TH, Choe Y, Ham A, Choi JH (2013). Functional characterization of genetic variations in the MDR3 promoter. Biochem Biophys Res Commun.

[40] Jeong HJ (2016). Cerebellar Development in Preterm Infants at Term-Equivalent Age Is Impaired after Low-Grade Intraventricular Hemorrhage. J Pediatr.

[41] Ball G (2010). An optimised tract-based spatial statistics protocol for neonates: applications to prematurity and chronic lung disease. Neuroimage.

