# Supplementary material for: Functional variation of SHP-2 promoter is associated with preterm birth and delayed myelination and motor development in preterm infants
Source: Sci Rep. 2017 Jul 20;7:6052. doi: 10.1038/s41598-017-06401-x (PMC5519743; doi:10.1038/s41598-017-06401-x)
Supplement: Supplementary file 1 — Supplementary Information [file 41598_2017_6401_MOESM1_ESM.pdf]

Supplementary Information

For

Functional variation of *SHP-2* promoter is associated with preterm birth and delayed myelination and motor development in preterm infants

So-Yeon Shim[1], Hye Jin Jeong[2], Hyo Jin Park[3], Eun Young Kwon[3], Bo Min Kim[3], Yang Ji Choi[3], Youn-Hee Choi[4], Su Jin Cho[1], Ji Ha Choi[3,*], Eun Ae Park[1,*]

[1]Division of Neonatology, Department of Pediatrics, School of Medicine, Ewha Womans University, Seoul, Korea

[2]Neuroscience Research Institute, Gachon University, Incheon, Korea

[3]Department of Pharmacology, Tissue Injury Defense Research Center, School of Medicine, Ewha Womans University, Seoul, Korea

[4]Department of Physiology, Tissue Injury Defense Research Center, School of Medicine, Ewha Womans University, Seoul, Korea

[*]corresponding authors: pea8639@ewha.ac.kr or jihachoi@ewha.ac.kr

***Correspondence to**

Eun Ae Park, MD, PhD

Division of Neonatology, Department of Pediatrics, School of Medicine, Ewha Womans University

1071 Anyangcheon-ro, Yangcheon-gu, Seoul, 07985, Korea

Phone: +82-2-2650-5574; Fax: +82-2-2653-3718; E-mail: pea8639@ewha.ac.kr

Ji Ha Choi, MD, PhD

Department of Pharmacology, Tissue Injury Defense Research Center, School of Medicine, Ewha Womans University

1071 Anyangcheon-ro, Yangcheon-gu, Seoul, 07985, Korea

Phone: +82-2-2650-5746; Fax: +82-2-2653-8891; E-mail: jihachoi@ewha.ac.kr

Supplementary Figure 1. Characteristics of the study subjects.

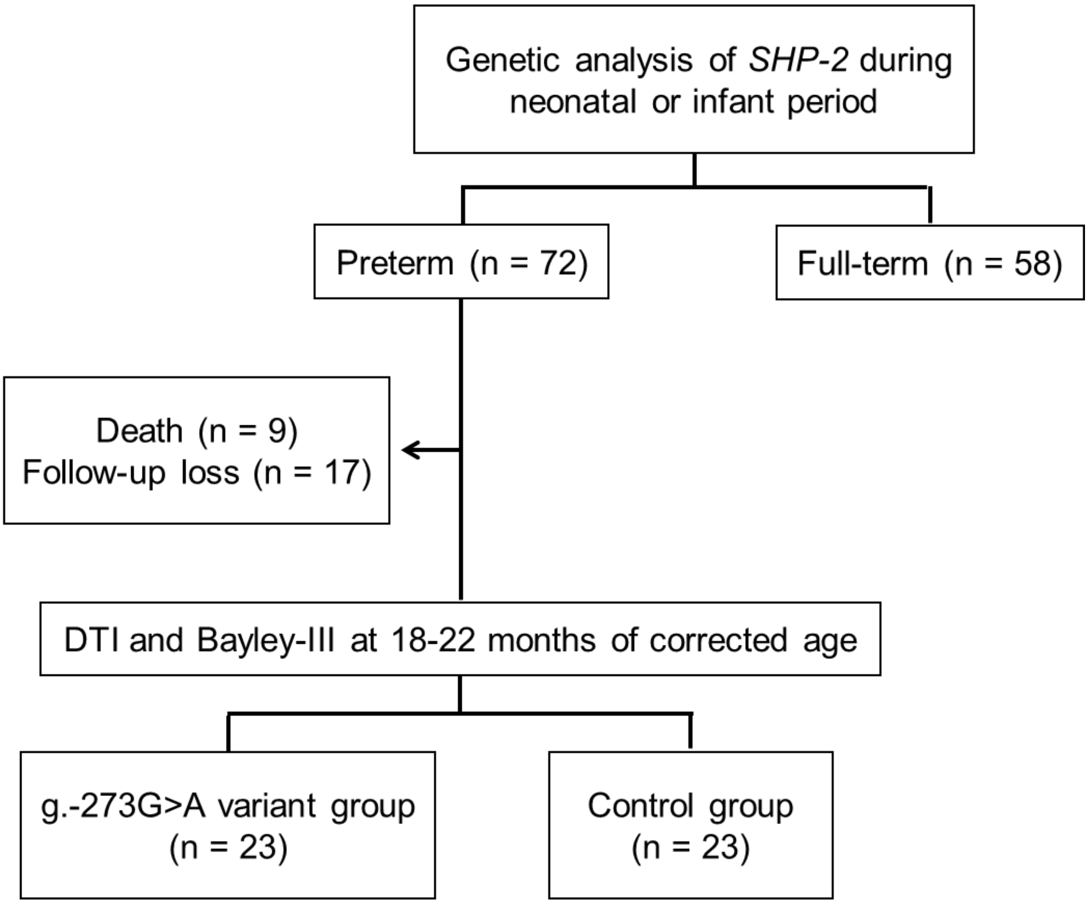

Supplementary Table S1. Analysis of regions affected by *SHP-2* variation, g.-273G>A

MNI coordinates			Anatomical location
x	y	z	
-42	-65	51	Superior parietal lobule
-40	-62	45	Inferior parietal lobule
-38	-66	31	Angular gyrus
21	-37	31	Medial frontal gyrus
20	-36	31	Cingulate gyrus
-54	-52	27	Supramarginal gyrus
-32	-33	12	Transverse temporal gyrus
-43	-21	9	Insula
-49	-20	4	Superior temporal gyrus
-29	-63	-2	Lingual gyrus
36	-15	-5	Claustrum
-48	-11	-14	Middle temporal gyrus
19	-68	43	Precuneus

MNI, Montreal neurological institute

Supplementary Table S2. Frequency of g.-273G>A from diverse populations

Population	Minor allele frequency	Population	Minor allele frequency
ASW	0.156	CHB	0.320
CEU	0.111	JPT	0.226
CDX	0.204	YRI	0.157

Data was obtained from 1000 Genomes Project (phase 3).

ASW (Americans of African Ancestry in SW USA), CEU (Utah Residents), CDX (Chinese Dai in Xishuangbanna, China), CHB (Han Chinese in Beijing, China), JPT (Japanese in Tokyo, Japan), YRI (Yoruba in Ibadan, Nigeria).

Supplementary Table S3. Oligonucleotides used in this study

Primes for *SHP-2* promoter cloning[a] (-361 to +87; 448 bp)	
Sense (XhoI site)	5'-ATG C**CT CGA G**GA AGC AAG GAT GCT TTG GAC-3'
Antisense (NheI site)	5'-AAT **GCT AGC** CTT CCG GAC GGG GCT AAC-3'
Primers for *SHP-2* mutagenesis PCR[b]	
g.-317C>T	5'-CCT CCG CGG AG**T** CCC CGC GCT GC-3'
g.-273G>A	5'-GGT CCT CCG CTG AC**A** GGA AGC AGG AAG TG-3'
P559S	5'-GTG GAG ATC AGA GC**T** CTC TCC CGC CTT GT-3'
Oligonucleotides for EMSA	
Wild-type (g.-317C)[b]	5'-GCC TCC GCG GAG **C**CC CCG CGC TGC C-3'
Variant (g.-317T)[b]	5'-GCC TCC GCG GAG **T**CC CCG CGC TGC C-3'
Consensus NF-κB[c]	5'-AGT TGA **GGG GAC TTT CCC** AGG C-3'
Wild-type (g.-273G)[b]	5'-TCC TCC GCT GAC **G**GG AAG CAG GAA G-3'
Variant (g.-273A)[b]	5'-TCC TCC GCT GAC **A**GG AAG CAG GAA G-3'
Consensus GABPα[c]	5'-AGA GGA TTG TGG GAC **CGG AAG CGG AAG** AGA AGC-3'
Mutant consensus GABPα[d]	5'-AGA GGA TTG TGG GAC **TAC** AAG **TAC** AAG AGA AGC-3'

[a] The restriction endonuclease sites were marked by bold-faced letters.
[b] The SNP sites were marked by bold-faced letters.
[c] The consensus sequences of transcription factors were marked by bold-faced letters.
[d] The changes in consensus sequences were marked by bold-faced letters.
